# Design and Implementation of a postgraduate curriculum to support Ethiopia's first emergency medicine residency training program: the Toronto Addis Ababa Academic Collaboration in Emergency Medicine (TAAAC-EM)

**DOI:** 10.1186/s12909-018-1140-3

**Published:** 2018-04-06

**Authors:** Nazanin Meshkat, Sisay Teklu, Cheryl Hunchak, On behalf of TAAAC-EM and the Global Health Emergency Medicine (GHEM) organization at the Division of Emergency Medicine, University of Toronto

**Affiliations:** 1 Division of Emergency Medicine, Faculty of Medicine, University of Toronto, Toronto, Canada; 2 Department of Emergency Medicine, University Health Network, Toronto, Canada; 3 College of Health Sciences, Addis Ababa University, Addis Ababa, Ethiopia; 4 Department of Family and Community Medicine, University of Toronto, Toronto, Canada; 5 Department of Emergency Medicine, Mount Sinai Hospital, Toronto, Canada

**Keywords:** Emergency medicine curriculum, Graduate medical education, International health, Global health, Capacity building

## Abstract

**Background:**

To design and implement an emergency medicine (EM) postgraduate training curriculum to support the establishment of the first EM residency program at Addis Ababa University (AAU).

**Methods:**

In response to the Ethiopian Federal Ministry of Health mandate to develop EM services in Ethiopia, University of Toronto EM faculty were invited to develop and deliver EM content and expertise for the first EM postgraduate residency training program at AAU. The Toronto Addis Ababa Academic Collaboration-EM (TAAAC-EM) used five steps of a six-step approach to guide curriculum development and implementation: 1. Problem identification and general needs assessment, 2. Targeted needs assessment using indirect methods (interviews and site visits of the learners and learning environment), 3. Defining goals and objectives, 4. Choosing educational strategies and curriculum map development and 5. Implementation.

**Results:**

The needs assessment identified a learning environment with appropriate, though limited, resources for the implementation of an EM residency program. A lack of educational activities geared towards EM practice was identified, specifically of active learning techniques (ALTs) such as bedside teaching, simulation and procedural teaching. A curriculum map was devised to supplement the AAU EM residency program curriculum. The TAAAC-EM curriculum was divided into three distinct streams: clinical, clinical epidemiology and EM administration. The clinical sessions were divided into didactic and ALTs including practical/procedural and simulation sessions, and bedside teaching was given a strong emphasis. Implementation is currently in its seventh year, with continuous monitoring and revisions of the curriculum to meet evolving needs.

**Conclusion:**

We have outlined the design and implementation of the TAAAC-EM curriculum; an evaluation of this curriculum is currently underway. As EM spreads as a specialty throughout Africa and other resource-limited regions, this model can serve as a working guide for similar bi-institutional educational partnerships seeking to develop novel EM postgraduate training programs.

**Electronic supplementary material:**

The online version of this article (10.1186/s12909-018-1140-3) contains supplementary material, which is available to authorized users.

## Background

The burden of trauma, infectious and chronic diseases is high in Ethiopia. Due to poor health care access for myriad reasons including long distances to hospitals, limited transportation, limited ability to pay for care and a severe human health resources (HHR) shortage, patients often present for care in extremis, at late stages of illness and post-injury, making quality emergency care provision imperative.

At the turn of the twenty-first century, as in most low and middle-income countries (LMIC), emergency care was provided in rooms within separate outpatient departments (e.g. pediatrics, obstetrics gynecology, surgery and medicine), similar to the pre-emergency medicine (EM) days in North America (Kirsch et al.) [1]. Pre-hospital services, triage systems, resuscitation rooms and emergency protocols were non-existent. Training, staff and resuscitation skills were minimal. Medical trainees and nurses without dedicated EM training managed emergent care cases. As a result, the Ethiopian medical system lacked the capacity to respond effectively to the needs of emergency patients leading to high morbidity and mortality. It is estimated that 10–15% of deaths in low and middle-income countries (LMIC) occur during emergency care [2].

The development of EM services in low-income countries is a complex undertaking and requires a multi-faceted approach, with the training of quality health care workers a crucial and challenging aspect to be addressed. Advocacy for capacity building in EM services culminated in the support and call for its development by the Ethiopian Federal Ministry of Health (FMOH) in the 1990’s [3].

Addis Ababa University (AAU), a well-established academic institution in Ethiopia, formed multiple north-south partnerships to collaborate and mentor educational capacity building. These collaborations have ranged from short-term training programs to long-standing partnerships. One such longstanding partnership is the Toronto Addis Ababa Academic Collaboration (TAAAC), a bi-institutional academic collaboration between the University of Toronto (UT) and AAU. TAAAC was established with the goal of building capacity at AAU in the medical specialties and in other health professional programs. Its mandate is to address educational gaps by providing trainers and training materials to medical residencies and sub-specialty programs at AAU. The TAAAC model for medical specialties consists of three one-month in-country teaching trips per year staffed by two UT faculty members and one senior resident. The UT teams work closely with their Ethiopian counterparts to longitudinally train Ethiopian postgraduate medical trainees.

A subsection of the TAAAC program is TAAAC-Emergency Medicine (TAAAC-EM). TAAAC-EM, amongst other partners, was invited to assist AAU in establishing the first-ever EM residency program in Ethiopia. In this paper, we describe the design and implementation of the UT curriculum contributions to the EM postgraduate residency training program established at Tikur Anbessa Specialized Hospital (TASH) in Addis Ababa, Ethiopia. To our knowledge, this is the first paper describing the design and implementation of an EM curriculum in a LMIC setting as part of a bi-institutional partnership. In this manuscript, we do not describe the development of other elements of the TAAAC-EM program nor the broader planning and implementation of Ethiopian EM services [4, 5].

## Methods

We used the six-step approach outlined by Kern DE et al. to guide curriculum development and implementation. In this manuscript we provide an outline of the first five steps, outlined below [6]. The final step, evaluation and feedback, is currently underway and will be reported elsewhere.

### Step 1: Problem identification and general needs assessment

As outlined above, the Ethiopian FMOH mandated the development and implementation of EM services in Ethiopia in response to the identified health systems need [3]. In 2006, an Emergency Medicine Task Force (EMTF) was struck at AAU comprising faculty appointees from medicine, surgery, obstetrics-gynecology, anesthesia and pediatrics [5]. These physicians championed the country’s first EM postgraduate training program and oversaw the consolidation of the TASH medical and surgical outpatient departments into a single emergency department (ED). EM faculty from UT were then invited to develop and deliver the EM-specific content of the postgraduate residency training program due to the absence of in-country board-certified Ethiopian EM physicians.

### Step 2: Targeted needs assessment

A needs assessment was conducted in 2009 using indirect methods (interviews and site visits) of the learners and learning environment to identify educational needs, opportunities and challenges in the design and implementation of an EM curriculum at AAU. Two experienced UT EM physicians conducted the interview and site visit components of the needs assessment during a two-week visit to TASH. The specific components of the needs assessment are described below.

### Trainee, staff and site interviews

A series of interviews of trainees (medical students and non-EM residents) and ED staff (nurses and general practitioners) at the new TASH ED were conducted to delineate the following information: 1) medical training prior to residency, 2) existing educational infrastructure at the postgraduate level, 3) learner perceptions and needs, 4) current evaluation framework, 5) disease burden and 6) disease-based management and perceived gaps.

To corroborate and strengthen the findings of the interviews, medical, surgical, pediatric and obstetric-gynecology wards and rounds at TASH were visited to identify off-service educational activities and requirements. Visits to the outpatient departments at other hospitals were also conducted to interview staff and understand the broader context of practice in Addis Ababa.

### Emergency department staff, flow and logistics

The TASH ED flow and protocols were reviewed to gain an understanding of the ED logistics and available resources including staffing, registration, triage, charting, equipment and medication supply/stocking and the context of the educational milieu where resident training would ensue. This information is summarized in Table 1. Availability of consulting services and outpatient departments was determined. Ancillary departments including the laboratory, radiology department and blood bank were visited to identify and understand barriers to patient care and flow. The triage intake form at the TASH ED was reviewed with the triage nurse on a daily basis to further understand the demographics and burden of disease and to help characterize the context-specific educational needs of the incoming residents (Table 2).

**Table 1:** Overview of available equipment, supplies, ancillary departments, inpatient services and outpatient clinics at TASH, 2009

*Equipment*	
*Available to all ED patients:* Defibrillator, monitors, ECG, oxygen cylinders, nasal prongs, blood pressure cuffs, thermometers. *Available if purchased by patient:* IV cannula, IV solutions (normal saline and ringers lactate), lumbar puncture trays, chest tube trays, gauze, gloves, casting material, medications *Not available:* Ventilator, otoscope, ophthalmoscope, glucometers, portable oxygen monitors, slit lamp.	
*Ancillary Departments*	
Laboratory services	Available tests: complete blood count, chemistry (Cl and HCO3 not routinely done), INR and PTT, liver enzymes, blood smears for parasitology, urine tests, gram stain and cultures, CSF analysis, sputum cultures and acid fast bacilli. No blood gas analysis capability. Limitations: After hours blood test availability. Long turnaround times (e.g. CBC and chemistry were 6 and 4 h, respectively). Blood cultures were limited by a shortage of blood culture bottles.
Blood Bank	Available products: Packed red blood cells and platelets. Occasional fresh frozen plasma and cryoprecipitate. Blood products are screened for VDRL, HIV, Hepatitis B and Hepatitis C. Limitations: chronic shortages
Radiology services	Available: X-rays, ultrasound. Limitations; Not available after hours, delayed turnaround time for typical x-ray films, inadequate x-rays, no functioning CT in the hospital (available at cost at private centers)
*Inpatient services*	
6 bed medical intensive care unit 6 bed surgical intensive care unit Internal medicine with subspecialties (such as infectious diseases, endocrinology, gastroenterology, nephrology, neurology, rheumatology etc) Orthopedics General Surgery Urology Neurology (a new fellowship) Neurosurgery (a new specialty) Pediatrics Obstetrics and Gynecology. Cardiology and interventional cardiology, cardiac surgery, vascular surgery, plastics surgery and dialysis capability were only available at private hospitals at significant expense to the patient	
*Outpatient Clinics*	
Tuberculosis clinic HIV/Infectious disease clinic Internal Medicine clinics (endocrinology, gastroenterology, nephrology, neurology, rheumatology) Surgical clinics (urology, thoracic, general surgery, orthopedics fracture clinic) Resident’s follow up clinic – surgical and medical

**Table 2:** Web search for free open-access EM-specific educational resources developed and implemented in LMIC settings

Emergency Medicine Organization Websites
Canadian Association of Emergency Physicians (CAEP)
American College of Emergency Physicians (ACEP)
International Federation of Emergency Medicine (IFEM)
Society for Academic Emergency Medicine (SAEM)
Emergency Medicine Society of South Africa (EMMSA)
African Federation of Emergency Medicine (AFEM)
European Society of Emergency Medicine (EuSEM)
World Association for Disaster and Emergency Medicine (WADEM)
Asian Society of Emergency Medicine (ASEM)
Global health sites and open access sites
Life in the Fast Lane
Consortium of Universities for Global Health (CUGH)
John Hopkins Global Health Center

### Step 3: Goals and objectives

The UT delegation met the EMTF to identify curricular goals and objectives and to delineate the accreditation requirements for the implementation of an EM postgraduate residency program. A literature search was conducted using the following keywords: “emergency medicine curriculum”, and “low-and-middle-income countries”, or “low resource settings” or “developing countries” to identify existing resources for guidance in the development of curricular goals and objectives in a LMIC context.

### Step 4: Educational strategies (curriculum development)

A curriculum working-group of EM physicians at UT were tasked with the development of the TAAAC-EM curriculum content and delivery methods for the three-year AAU EM postgraduate residency training program. Specifically, the curriculum outlined in this paper was developed for implementation by UT teaching teams following the overall TAAAC program model: three one-month in-country teaching trips per year, staffed by two UT EM faculty members and one senior EM resident. Off-service rotations comprise the rest of the curriculum under the purview of the EMTF and are not outlined in detail here. A curriculum map was developed.

A web search was conducted in 2010 to identify free open-access EM-specific educational resources developed and implemented in LMIC settings that could be used and/or adapted to the Ethiopian context. We searched the websites of EM organizations’ general webpages and global health web pages, where available, for open access educational modules geared towards LMIC (Table 2).

### Step 5: Implementation

The curriculum map was shared with both the TAAAC-EM executive and the EMTF, and was subsequently implemented beginning in 2010. The curriculum was reviewed and refined by the curriculum working-group following each one-month in-country teaching trip. Feedback was obtained from Ethiopian trainees, the EMTF and returning UT faculty/residents. The UT curriculum co-directors (NM, CH) made regular on-site visits throughout the first three years of curriculum implementation to teach and to assist in implementation.

## Results

### Step 1 and 2: Needs assessment findings

The trainee and staff interview components of the needs assessment are summarized below.

### Trainee and staff interviews, and site interviews

1. Medical training prior to residency

Medical school is 6 years in length with the early years consisting of didactic, lecture-based teaching, followed by a clinical clerkship model. After completion of medical school, it is obligatory to do a 2-year placement in an under-serviced area, where physicians are referred to as general practitioners (GP). Once the placement is completed, physicians are eligible to enter specialty training ranging in duration from 3 to 5 years. Importantly, we identified that no EM training was provided to medical students or GPs.

2. Existing educational infrastructure at the postgraduate level

Our interviews identified important educational gaps at the departmental and institutional levels. At the ED level, educational activities geared specifically towards EM practice were limited and primarily consisted of morning meetings discussing ED cases seen the night prior. EM-specific seminars, practical/procedural training sessions, simulation training, morbidity and mortality rounds and journal clubs were lacking. At the institutional level, apart from formal weekly didactic lectures, the education system also did not utilize the aforementioned active learning sessions such as simulation and practical/procedural training sessions. Significantly, a culture of minimal bedside supervision and teaching was recognized as a by-product of a shortage of the physician workforce struggling to meet clinical demands (further details below).

3. Learner perceptions and needs

English was the main language used for medical teaching and was not identified as a major barrier. Trainees and staff identified the need for improved educational activities including bedside teaching. Basic research capacity and quality improvement expertise were also identified by both the EMTF and other staff/trainees as a significant educational gap.

4. Evaluation framework

In both the ED and at the institutional level, a lack of daily and end-of-rotation evaluation and feedback mechanisms were identified. Formal evaluative tools using the traditional examination model with both written and practical exams were commonly used.

5. Disease burden

The most common conditions seen at TASH were trauma from road traffic accidents, work-related accidents and street violence, infectious diseases including HIV/AIDS and tuberculosis, diabetic ketoacidosis and manifestations of rheumatic heart disease including heart failure and atrial fibrillation. These findings were corroborated with the triage record review. The median age of ED presentation was 30 years and trauma presentations comprised 49.2% of 125 visits.

6. Disease-based management

Interviews of the residents, interns and medical students staffing the ED revealed a non-uniform knowledge of EM practices including trauma and resuscitation protocols, airway management, acute cardiac life support, sepsis management and pain management, among many others.

### Emergency department staff, flow and logistics

#### Staffing

At the time of our needs assessment, there were no dedicated emergency physicians working in the ED. Physician coverage consisted of junior medical and surgical residents and interns, and occasionally GPs. Senior residents and staff were not expected to be present in the ED, but to provide backup to the junior learners. The assignment of GPs to the medical and surgical sides of the ED had been implemented one year prior to the needs assessment. GP coverage was limited to daytime hours during the week; after hours, the interns and residents oversaw patient care. Although there was on-call staff coverage, interns and residents rarely communicated with staff.

Nursing coverage was provided 24 h per day. At the time of the needs assessment, nurses had received one month of training to work in the ED. The number of nurses per shift was limited relative to the number of patients.

#### Flow and logistics

The majority of patients presented to the ED as walk-ins. There was a paucity of public ambulance services in Addis Ababa, a situation that is worse in the rest of the country. A few private ambulance services exist. The TASH ED accepts adult patients; all pediatric and obstetric cases are diverted to their respective outpatient departments at triage.

### Step 3: Goals and objectives

The EMTF authored and provided the UT EM delegation with a document called “Curriculum for Postgraduate study on Emergency Medicine” (Additional file 1)*.* In this document, core competencies of a 3-year EM postgraduate specialty program were outlined. The concurrent literature search did not reveal any documents relevant to the development of an EM postgraduate curriculum in LMIC; however a draft of a relevant EM curriculum written by the International Federation of Emergency Medicine (IFEM) was identified, which was subsequently published [7]. The goals and objectives outlined in this draft document and the EMTF document were closely reviewed. The competencies and goals and objectives laid out in the EMTF document were endorsed and used as a template for the curriculum map.

In addition to the overarching goals and objectives set forth in the EMTF document, the following core goals were identified by the UT curriculum working-group:

1. To provide context-specific educational content to learners to empower them to effectively manage the emergent medical needs of the local patient population
2. To foster experts in EM in knowledge and competence, and to enable translation of competencies to the bedside through ongoing supervision and role-modeling
3. To create graduates with a knowledge in EM administration and research to lead the development of EM in Ethiopia
4. To train the next cohort of trainers through effective role-modeling and mentoring, and by fostering the importance of medical education

### Step 4: Educational strategies (curriculum development)

Based on the findings of the needs assessment and the EMTF’s desired competencies for Ethiopia’s first EM physicians, a curriculum map was devised to supplement the AAU EM residency program curriculum. The TAAAC-EM curriculum was divided into three distinct streams: clinical, clinical epidemiology and EM administration.

The clinical sessions were divided into didactic and active learning sessions including practical/procedural and simulation sessions. Examples of topics covered in the practical/procedural sessions included casting, ECG interpretation, radiologic interpretation, chest tube insertions, airway management, etc. The didactic clinical sessions were divided into blocks (e.g. cardiology, toxicology, infectious diseases) with active learning sessions aligned to meet the objectives of each block. Learning objectives and reading materials from a provided standard EM textbook were outlined in the curriculum map and provided to residents in advance of the teaching months. An example of the curriculum map is provided in Additional file 2.

The TAAAC-EM curriculum was organized into an annually repeating junior (PGY1) lecture series comprising clinical and clinical epidemiology topics and a bi-annually repeating senior (PGY2–3) lecture series comprising clinical and administration topics. The clinical topics including the didactic, simulation and practical sessions were organized to introduce foundations of EM in the junior year of the residency (e.g. airway management, resuscitation principles etc.). This was in recognition of the fact that junior residents are responsible for unsupervised patient management at the outset of their training. In senior years, the clinical topics focused on core competencies of EM and built on competencies introduced in the junior years. All clinical topics were chosen based on the context-specific diseases identified during the needs assessment with a strong focus on management of trauma and infectious diseases. Further, the teaching of clinical topics was catered to the available resources (e.g. the eye exam did not rely on a slit lamp as there was none available).

The search for EM-specific educational resources developed and implemented in LMIC did not yield any open access or high quality resources at the time of the search. During the first four years, specific lecture materials were assigned by the curriculum co-directors to be developed by teaching team members. Specific lecture objectives and epidemiology at the TASH ED were communicated to authors to ensure contextual and trainee-level relevance of the delivered teaching materials. The authors, chosen based on their interest and expertise in global health, were responsible for conducting literature searches to identify relevant material for practice in Ethiopia and LMIC. Following teaching trips, the curriculum co-directors reviewed and archived the developed lectures for future use by later teaching teams. Subsequent UT teachers benefitted from the provision of pre-made lecture materials but remained responsible for content updates and ongoing improvements based on compiled AAU EM resident evaluation feedback. These modules undergo peer-review and are published on an open access website [8].

Lecture-specific, rotational and teacher evaluations were adapted from UT postgraduate medical education documents and reviewed with the EMTF. An EthioMEDs framework (Additional file 3), previously adapted from the CanMEDS framework by the TAAAC program, was used to structure the monthly resident rotation evaluations [9]. Individual resident mid-term and end-of-rotation evaluations were completed by UT EM faculty teachers during teaching trips and then shared with and archived at AAU by the EMTF.

### Step 5: Implementation

The curriculum map was approved by the TAAAC-EM executive and EMTF.

It was implemented over a recurring 12-trip (four year) teaching template. Lectures and practical teaching sessions were scheduled to take place three half-days per week during in-country teaching trips, with the rest of the time dedicated to full time bedside teaching in the ED. During teaching trips, UT faculty and senior resident teachers led morning patient rounds, reviewed new cases in the ED, assisted with running codes and resuscitations, delivered the scheduled didactic and practical teaching sessions and led a journal club. Responding to the findings of the needs assessment, bedside teaching and clinical role modeling were given significant emphasis in the curriculum implementation, allowing ample time for UT EM staff to serve as hands-on bedside teaching consultants.

Prior to TAAAC-EM trip departures, EM faculty and resident teachers undergo multiple trip briefings. These include a mandatory session led by the TAAAC founder, as well as two specific TAAAC-EM briefings during which a detailed description of expected teaching duties, review of teaching materials, and evaluation forms to be completed are outlined by the curriculum co-directors. An experienced team leader accompanies each teaching trip to Addis Ababa to facilitate introductions, liaise with the EMTF and orient the teaching team to their educational and clinical duties.

The curriculum co-directors both participated as teaching faculty during the early years of the program for a total of 6 one-month on-site teaching visits. Based on continuous monitoring and revisions, the curriculum schedule and content necessarily evolved to meet the changing needs of the EM training program and residents over time. Two key examples of this included a shift to a ‘half-day back’ educational model for senior EM residents as the roster of residents grew to a full complement of residents in PGY 1 to 3, and the incorporation of a point-of-care ultrasound (PoCUS) curriculum into both the clinical and practical curriculum streams with the growing relevance and educational demand for PoCUS training.

Within the first year of curriculum implementation, the need for distance-bridging initiatives was identified by both the AAU EM residents and UT teaching team members. Two such initiatives were implemented: 1) a formal faculty-resident mentorship program, and 2) scheduled monthly videoconferencing sessions.

At the time of manuscript preparation, a total of 22 TAAAC-EM teaching trips have been delivered. During these teaching trips, 239 didactic lectures, 75 practical sessions and videoconferencing sessions, 24 simulation sessions and 22 journal club sessions have been conducted. Twenty-one Ethiopian residents have graduated from the program, with thirteen residents set to graduate in February 2018, and another 44 are currently in training. 5 graduates are working as ED staff at TASH, with 15 actively working at other centers in Ethiopia. 34 UT faculty and 22 senior residents have travelled to Ethiopia to teach during TAAAC-EM teaching months. Twenty-five modules are peer-reviewed and published online, with 15 others undergoing peer-review. (8) An evaluation of the TAAAC-EM curriculum, as part of a formal TAAAC-EM program evaluation, is currently underway.

## Discussion

We have outlined the design and implementation of the TAAAC-EM longitudinal curriculum which provides EM-specific content for the first postgraduate EM residency training program established in Ethiopia. The EM program at AAU is currently thriving in its seventh year and is the second longest-standing program on the African continent, with South Africa and Tanzania. A sixth cohort of graduates will join a growing cadre of Ethiopian emergency physicians in January 2019. New graduates are filling staff positions in EDs across Addis Ababa, assuming leadership positions and leading similar initiatives in other parts of Ethiopia.

Severe human health resource shortages in LMICs is a pressing and complex problem. Africa disproportionately shoulders this crisis, carrying 24% of the global burden of disease with only 3% of the global health care workforce to address it [10]. Short term training programs, while infinitely logistically easier to implement, do not address capacity building and health leadership development, both of which are required to train and retain quality health workers and to improve patient outcomes in the long term. TAAAC-EM is a robust bi-institutional partnership, and the design and implementation of the TAAAC-EM curriculum has proven successful, feasible and sustainable within the organizational model of TAAAC. In an era where the EM specialty is growing globally and exponentially, the model provided in this manuscript may be considered for future bi-institutional partnerships in lieu of short-term courses. The findings of the curriculum evaluation currently underway can delineate future efforts to improve the program, and to inform other educational endeavors.

Inevitably, challenges have been encountered along with the successes. The creation of novel, context-specific teaching materials for low-resource EM settings has required a significant and ongoing academic resource investment, particularly during the first three years of the program. At the core, striking an optimal balance between teaching residents both high and low-resource setting approaches to patient care is a continual struggle. The development of context-specific educational materials by outside experts also has its inherent limitations. Further study is required to determine the success of the TAAAC-EM curriculum, designed and implemented by non-Ethiopians for Ethiopians, in enabling Ethiopian physicians to meet the challenges of EM practice in their context. As expertise grows in Ethiopia, greater involvement by local leaders and credentialing organizations will be essential to delineate competency requirements to meet societal needs and improve patient outcomes.

The intermittent nature of TAAAC-EM teaching trips and communication challenges during offline months has been identified as an ongoing challenge in a nascent field where, when the visiting team leaves the country, so does the EM clinical expertise. This has contributed to labile morale amongst Ethiopian residents. To bridge these challenges various attempts have been made including the aforementioned mentorship program and videoconferencing sessions; however, poor internet connectivity remains a significant impediment, especially with respect to videoconferencing. As the cohort of Ethiopian EM graduates grows, their engagement and leadership in educational initiatives should be fostered to continue to build local faculty capacity. Accordingly, a graded transition of TAAAC-EM teaching responsibilities to AAU EM graduates and new faculty is underway.

There is a growing awareness in medical education of the importance to align physician competence with societal needs. An inherent weakness in our needs assessment, due to funding, time and logistical constraints, was the narrow scope. We focused the needs assessment at TASH where the epidemiology of disease, resources and infrastructure are markedly different from other cities and in the vast rural areas in Ethiopia. Hence, the needs assessment findings and subsequent curriculum development may not align with Ethiopia’s societal needs at large. This is a fundamental consideration for the future implementation of EM in Ethiopia. As graduates of the EM program assume leadership positions across Ethiopia and beyond, the engagement of local populations to best address patient acute care needs is paramount.

As competency-based training programs gain momentum in medical education, future iterations of our curriculum will require careful attention to outcomes-based curriculum delivery incorporating multifaceted assessments to ensure that outcomes are met [11–13]. This is currently underway with the point of care ultrasound aspects of the curriculum and will expand further in time. Finally, novel approaches and assessments must be implemented to ensure that competencies are achieved in roles other than the EthioMEDs intrinsic medical expert role.

In conclusion, the TAAAC-EM partnership has been successful at developing and delivering an EM postgraduate training curriculum to supplement the overall AAU EM residency program curriculum, and is one of the first and most mature on the African continent. As a thoughtful model for sustained north-south medical education partnerships, it can serve as a roadmap for other similar initiatives on the African continent and elsewhere.

## Additional files

Additional file 1: Curriculum for Post Graduate Study on Emergency Medicine. (PDF 236 kb)

Additional file 2: Teaching Trip 1–12 Templates at a glance. Sample Detailed Teaching Template – Trip 3. (PDF 166 kb)

Additional file 3: Brief Explanation of CanMEDS/EthioMEDS roles. (PDF 84 kb)

## Abbreviations

AAU: Addis Ababa University
ALTs: Active learning techniques
ECG: Electrocardiogram
ED: Emergency department
EM: Emergency medicine
EMTF: Emergency Medicine Task Force
FMOH: Ethiopian Federal Ministry of Health
GHEM: Global Health Emergency Medicine
GP: General practitioner
HHR: Health human resources
HIV/AIDS: Human immunodeficiency virus/acquired immune deficiency syndrome
IFEM: International Federation of Emergency Medicine
LMIC: Low and middle-income countries
PGY: Postgraduate year
PoCUS: Point-of-care ultrasound
TAAAC: Toronto Addis Ababa Academic Collaboration
TAAAC-EM: Toronto Addis Ababa Academic Collaboration in Emergency Medicine
TASH: Tikur Anbessa Specialized Hospital
UT: University of Toronto
