# Supplementary material for: Design and Implementation of a postgraduate curriculum to support Ethiopia's first emergency medicine residency training program: the Toronto Addis Ababa Academic Collaboration in Emergency Medicine (TAAAC-EM)
Source: BMC Med Educ. 2018 Apr 6;18:71. doi: 10.1186/s12909-018-1140-3 (PMC5889606; doi:10.1186/s12909-018-1140-3)
Supplement: Supplementary file 1 — Curriculum for Post Graduate Study on Emergency Medicine. (PDF 236 kb) [file 12909_2018_1140_MOESM1_ESM.pdf]

Curriculum for Post Graduate Study
On Emergency Medicine

Department Of Emergency Medicine,
Addis Ababa University, Medical Faculty

MARCH 2009

Table of Contents

Page

1. Back ground of the department-- 3

2. General Objectives-- 5

3. Specific Objectives-- 6

4. Programs of the department--6

5. Curriculum-- 6

5.1 Rationale of the curriculum-- 6

5.2 Graduate profile--- 7

5.3 Program requirement-- 8

5.4 Selection and sequencing of courses -- 9

5.5 Course continents and description --- 11

5.6 Teaching and learning methods and materials------------------------------- 14

5.7 Quality assurance -- 15

5.8 Resource profile -- 18

1. Background / Introduction

Emergency Medical Services (emergency medicine) (EMS) as a separate and district discipline is a recent development in the history of medical practice. Its originated three decades back in the USA and Canada.

Those who initially proposed the idea of EMS as a separate clinical discipline had long years of experience in clinical practice and have looked and analyzed the natural course of diseases, the final path way of disease progression leading to death and the similarity of final events leading to death in many disease conditions.

These and other similar objective observations substantiated their recommendation to establishing EMS as a separate clinical discipline. Since its introduction, EMS has been found to be more efficient and effective way of managing all acutely and critically ill patients. The goal of the discipline is to manage all critically sick emergency patients in one room equipped with all the necessary medical supplies and by one emergency team composed of emergency specialist doctors, nurses and other required staffs. The goal of EMS is to resuscitate and stabilize emergency cases, provide the required life saving medical and surgical interventions and ensure patients can be safely transferred to regular wards and clinics after certain period of stay in EMS unit/department. Emergency medical care provided in one room and by one organized specialist emergency medical care team since its origin, has shown to be more advantageous both in terms of saving and appropriately utilizing human and material resources and avoiding long interval arrival to intervention period that used to be spent for inter disciplinary consultation there by utilizing the critical, golden period fast to save patient's life.

The practice of EMS in its currently practiced form is recently being implemented in various parts of the world including Europe, Asia and some part of Africa.

In Ethiopia, where the traditional medical care facilities are limited in number and capacity, getting timely and optimal emergency care has been and still is practically difficult, if not impossible, even for those emergency patients with adequate resources to pay.

This together with increasing number of patients who need emergency care owing to prevalent diseases that can potentially lead to emergency condition and the high incidence of road traffic accident (one of the highest in the world) has led some teaching staff doctors of AAU-MF to look for a way of organizing emergency set up in Ethiopia and a number of efforts were made to introduce the idea of EMS and training in Ethiopia.

Data from institutions in other parts of the world with experience in EMS show that although there is no uniformity regarding priority area of interest in EMS and training, there appears to be similar objective, goal and organization of EMS and training. Some EMS centers give more emphasis on emergency cases due to trauma while others do emphasize more on medical emergencies like cardiovascular emergency diseases.

The task force (committee) established by AAU MF for the purpose of introducing EMS and training unit/department in Tikur Anbessa Specialized Hospital has revised the curricula for training EM specialist doctors of different institutions and also has tried to visit and get some experience from other institutions having reasonable experience and expertise in the field of EMS.

It is also believed by majority of practitioners that introducing EMS and training in our set up benefits the under graduate medical students training and trainee nurses by enabling them to have at least the minimum exposure on identification of patients who are in life threatening critical conditions, basic concepts on the principle of care for critically sick patients including basic resuscitation and stabilization mechanisms, also acquire skills on basic and advanced life support and important life saving procedures so that the deficiencies on medical professional graduates in this respect will be addressed.

Like the practice elsewhere and to facilitate the training process, it is believed that higher level EM training should be specialty graduate program by recruiting general practitioners with some year of experience in all major clinical disciplines.

The organization of EMS and training unit/department will not be markedly different from other clinical departments. But the issue of getting adequate EM specialist to implement the graduate training program will be one of the major limitations during the early phases of the training. We believe that, until the department stands on its feet and become self sufficient, collaboration and impute from other departments in the faculty will be of paramount importance. The specific emergency and other related topics out lined in this curriculum will be taken care of for the purpose of service and training by respective clinical and biomedical departments in accordance with the protocol designed by EMS and training department and schedule that will be notified to the departments before hand by allowing reasonable time to get prepared as this will be additional burden for the departments.

The overall activity will be monitored and followed by the Dean's Office together with EMS committee with close supervision of Academic Commission of the faculty where EMS and training unit will be represented.

2. General Objectives

To produce qualified emergency medicine specialists (Emergency Medicine Specialist Doctor) who can provide comprehensive emergency medical care in a set up with limited resources.

3. Specific Objectives

3.1 To train emergency specialist doctors that will provide optimal and comprehensive emergency care.

3.2 To train emergency specialist doctors with a broad scope of knowledge regarding basic principles of basic and advanced life support.

3.3 To enable trainees learn the triage, assessment, management and disposition of critically ill patients.

3.4 To get knowledge and be confident on prioritization principles, triage decision and surgical interventions.

3.5 To have optimal knowledge on disease epidemiology,

3.6 To get optimal skill on emergency medical and research, interpretation of Medical research.

4. Programs of the department

Programs of the department of emergency medicine are programs:

4.1 Under graduate medical students training on basic principles of emergency medical care.

4.2 MSC on emergency medicine nursing.

4.3 Graduate training for emergency medicine specialist doctors.

4.4 Paramedic and EMT for pre-hospital emergency care providers

5. Curriculum

5.1 Rational of the curriculum

The goal of the curriculum of AAU-MF Emergency Medicine specialty training is to make the graduating emergency medical specialist doctors competent enough to handle most emergency cases, to provide optimal and timely care in an organized and responsible manner.

Besides, high number of preventable deaths due to emergency medical conditions that we are having now, because of lack of skilled critical care professionals, lack of responsible and organized structure and lack of uniform

and applicable emergency management protocol, will be prevented when this curriculum is implemented. It is also believed that the presence of responsible department and higher level training for emergency cases handling will also provide important emergency data that will be utilized for future planning and priority setting.

5.2 Graduate Profile

Upon completion of the training the graduate will have acquired the following

Attitudes:-

1. Have and show at most respect and diligence to his/her profession
2. Devote his/her time, knowledge and power to the patient who need his/her help irrespective of the circumstance he/she is in at the time when his care is needed.
3. Understand and believe that the knowledge and skill acquired during the training saves patient life if used with concern responsible manner and dedication.
4. Use appropriately all the resources in the EMS unit with full responsibility
5. Have good relationship so that patients are cared and given optimal service.
6. Gives priority during providing emergency care based on urgency and degree of severity of specific case

Knowledge

1. Understand the normal physiologic, anatomic and biochemical make of human body.
2. Understand the pathophysiology of emergency disease conditions, natural course of diseases and be confident in predicting timely the possible consequences that will follow.
3. Have the knowledge of proper and fast assessment of critically sick patients and the principles of triaging system.

4. Have proper knowledge on basic and advanced life support, resuscitation and stabilization
5. Understand the protocols and guidelines of emergency medical care.
6. Be able to understand pharmacologic drugs used in emergency care; pharmacokinetics , drug interaction, side effects, and contra indications.

Skills

1. Have skills of conducting appropriate and fast evaluation of critically sick patient.
2. Perform emergency diagnostic tests.
3. Provide fast and effective basic and advanced life support, resuscitation and stabilization.
4. Have appropriate skills to perform the required emergency surgical Intervention.
5. Participate on training of medical professionals on EM.
6. Manage medical information and data systems.

5.3 Program Requirements

5.3.1 Academic admission requirement

5.3.1.1 MD degree or equivalent from a recognized

5.3.1.2 Satisfactory completion of internship at least in the four major clinical departments.

5.3.1.3 Passing the admission written examination and interview administered by the department with a score of 70 % and above.

5.3.2 Non-academic requirement

5.3.2.1 Should be registered and licensed to practice medicine in Ethiopia

5.3.2.2 Should be in adequate mental and physical health

5.3.2.3 Should be under the age of 40 at the time of application

5.3.3 Graduation requirement

The candidate should have

5.3.3.1 Successfully completed 36 months (3 years) of residency program.

5.3.3.2 Should submit a research work that has been rated as pass and forwarded with an endorsement that the requirement to sit for final qualifying examination has been partially fulfilled

5.3.3.3 Should have passed the final qualifying examination with score of 70% and above

5.3.4 Degree Nomenclature

Specialty certificate in Emergency Medicine

□□□□□□□ □□□□ □□h□□

5.4 Selection and Sequencing of Courses

5.4.1. **clinical rotations**

Rotation in Emergency Department and in the major and relevant Clinical Departments such a

- Anesthesiology
- M ICU
- Emergency Department
- Surgery
- medical ICU
- Ob-GY
- Orthopedics
- Pediatrics
- Radiology
- ENT
- Ophthalmology
- Psychiatry
- Dermatology

5.4.2 Sequencing of attachments

(a) YEAR I (12 Months)

Activities/ Rotation	Duration
- Anaesthesiology/OR	1 months
- M ICU	1 month
- Emergency Department	6 months(1 month paediatrics)
- Surgery	1 month
- Orthopedics	1 month
-Radiology	1 month
-vacation	1 month
**Total**	**12 months**

(b) YEAR II (12 Months)

Activities/ Rotation	Duration
- Emergency Department	6 months(1 month paediatrics)
- Ophthalmology	1 month
- Gyn-Obs	1 month
- Psychiatry	1 month
- ENT/Deantistry	1 month
- Pre Hospital/EMS	1 month
- vacation	1 month
**Total**	**12 months**

(c) YEAR III (12 months)

Activities/ Rotation **Duration**

- Emergency Department	7 months (1 month paediatrics)
- Research	1 month
- Surgery/orthopedics	1 month
- Anesthesiology and Surgical ICU	1 month
- study leave/vacation	2 months
**Total**	**12 months**

5.5. Course content and description

Rotation: Surgery/ Trauma

Institution: AAUMF department of Surgery and Orthopedics

Year of Residency: EM Year 1 and III

DESCRIPTION OF CLINICAL EXPERIENCE

During the surgical/trauma rotation, EM Resident is expected to participate actively in the initial evaluation, resuscitation, stabilization and definitive treatment of all forms of adult and pediatrics trauma.

The EM resident will be mentored, supervised and evaluated by faculties and senior residents like surgical resident of the same level. The resident will attend morning meetings, grand rounds, seminars and other departmental teaching activities. The resident will be assigned to night, weekend and holy day duty like other surgical residents of his/her level. The resident should be active in assisting and doing surgical procedures assigned by the surgical attending and the senior resident focusing on emergency surgery.

Rotation: Anesthesia and Critical care

Institution: AAUMF department of Anesthesia

Major OR and Surgical ICU

Year of Residency: EM Year 1

Duration in months: 8 weeks

Description of Clinical Experience

The EM resident will rotate in anesthesia department for 1 month during year one. The EM resident will be supervised by the anesthesia department consultants and senior residents.

The resident is expected to attend and present regularly morning meetings, seminars and other related teaching activities in the department of anesthesiology and will be assigned to night, weekend and holy day duty like other residents of his/her level.

The resident will do preanesthetic evaluations in consultation with the attending. The resident is expected to carry out his assignment in the OR and will be active participant in the SICU patient evaluation, monitoring and management.

Rotation: Medical ICU and Internal Medicine

Institution: AAU MF department of Internal Medicine

In patients ward, Medical ICU and Cardiac unit.

Duration in months: 4weeks each year

Year of training: EM-I

The resident will be mentored, supervised and evaluated by the respective attending and senior residents of the department. The EM resident is expected to carry out all assignments including actively involved in patient evaluation, treatment and doing procedures. The resident will attend and present morning sessions, seminars, grand rounds and other related teaching activities. The resident will be considered like other residents of the department of Internal Medicine of his/her level and treated as such during the time of the rotation. The resident will be assigned to night, weekend and holy day duty like other residents of his/her level.

Rotation: Obstetrics and Gynecology

Institution: AAUMF department of OB-GY

Duration in months: 4weeks each year

Year of training: EM-II

Description of Clinical Experience:

The EM resident, during the OB-GY rotation is expected to be an active member of the department of OB-GY. The resident is expected to attend and present morning meetings, case/death presentations, management sessions and other related teaching activities. The resident will be assigned to night, weekend and holy day duty like other residents of his/her level. The resident will assist and perform procedures described in this curriculum.

Rotation: Outpatient pediatrics/urgent care

Institution: AAU MF department of pediatrics and child health.

Duration in months: (4weeks each year)

Year of training: EM-I

Description of Clinical Experience

During pediatric rotation, the EM resident will be an active participant of the department of pediatric and child health. The resident will be assigned in the emergency pediatric units, will evaluate and manage cases in consultation with the pediatric senior residents. Morning meetings, seminars and other teaching activities will be most important part of their rotation. The resident will be assigned to night, weekend and holy day duties.

Rotation: Radiology

Institution: AAU MF department of Radiology

Duration in months: 1month (4weeks)

Year of training: EM-I

Description of Clinical Experience

During radiology rotation, the EM resident will be an active participant of the department of radiology. The resident will learn basic interpretation of x-ray films, ultrasound and CT scans application and interpretation in the context of emergency department. Morning meetings, seminars and other practical teaching activities will be most important part of this rotation. The resident will be assigned to night, weekend and holy day duties.

Rotation: Emergency Medical Services (Pre Hospital Care)

Institution: Addis Ababa City Fire and emergency department

Duration in month: 1 month

Year of training: II

Description of Emergency medical system Experience

The Emergency Medicine resident during pre hospital care rotation will be assigned at the central and satellite ambulance dispatch centers. The resident will have exposure in the over all administration of the pre hospital care. The resident will be updated with the relevant laws, guidelines and protocols. The head of the prehospital service will assign the resident to the different dispatch sites during the rotation.

5.6. Teaching and Learning Methods

The teaching and learning methods involves lectures, seminars, clinical rounds, case-presentations, and appraisal of scientific journals, simulation exercises of procedures, live demonstrations, group discussions, and brain storming sessions. The department will also make available facilities for self-directed learning and access to electronic library (e-journals, e-books and audio-visual aids).

5.6.1. Seminars: Once every week for 2 hours

5.6.2. Clinical Conferences: Once every month for 2 hours

5.6.3. Review of Admissions, Discharges and Death of Patients: Once every

week for 1 hour

5.6.4. Grand Rounds: Once every two weeks for 1 hour

5.6.5. Sessions to review scientific publications: Once every month for 1 hour

5.6.6. Seminars: Once every week for 2 hours

5.7 Quality Assurance _Maintaining Quality of the Program

5.7.1 Program Management and Monitoring Systems.

There will be under graduate, postgraduate, research and publication and other relevant committees that will be organized to carry out delegated responsibilities.

5.7.2 Formative and Summative assessments

The performance of each trainee is assessed as follows:

5.7.2.1 Formal Examination:

There shall be formal examination in year I
(at the end of basic science courses, part I phase I examination and basic clinical examination part I phase II) and year III (final exam). The student must pass the examination to be promoted to the next phase of the training and to be awarded the specialty certificate (diploma).

5.7.2.2 Progressive Assessment:

The responsible staff assess each trainee regularly and periodically at the end of each attachment with regard to trainees' knowledge, skills, attitude, and willingness to learn. The assessment will be compiled every six months. Trainees not performing to the standard will get feedback and advised to improve their performance.

5.7.2.3 Promotion With in the Program

5.7.2.3.1 Year I:

Medical Faculty's rules and regulation for clinical specialty programs will be used to promote a trainee within a program. To evaluate the trainee formal examination is conducted in the form of written, clinical and viva. In addition to the formal examination the academic staff assesses each trainee periodically. The marks shall be computed as follows:

Written:	30%
Viva:	10%
Practical/clinical	25%
OSCE	20%
Progressive Assessment	15%

1. A trainee should have a grade of 70% or above to be promoted to year II.
2. A candidate can repeat a year only once
3. A trainee that fails in the examination (grade less than 70%) shall be discussed at Department staff meeting and forwarded with recommendation to Medical Faculty Academic Commission (MFAC) for final decision.

5.7.2.3.2 Year II:

The Department and Medical Faculty Academic Commission (MFAC) should be satisfied by the performance of the trainee as evaluated by the progressive assessment to be promoted to the final year.

5.7.2.3.3 Year III:

The final year trainee shall compile and submit the completed research papers to the department for assessment six months before the scheduled final examination. Failure to submit on time and the research papers evaluated as substandard by the Department, the trainee will not be legible to sit for the final examination. The Department will forward the issue with recommendation to MFAC for appropriate action.

5.7.2.4 Dismissal from the Program

5.7.2.4.1 Disciplinary Dismissal:

Willful misconduct, a behavior not expected from a medical doctor that includes; negligence of duties and responsibilities that affect patients care, absence from work for non-substantial reasons, abuse of substance and alcohol, theft etc can be reasons for summary dismissal from the program. Such dismissal will be effected after receiving approval of the MFAC and the University's Graduate Council.

5.7.2.4.2 Academic Dismissal

According to the rules and regulations of Addis Ababa University Senate Legislation Article 3.6.8.1.

5.7.2.5 Final Examinations

5.7.2.5.1 Candidacy:

The trainee, to sit for the final qualifying examination he/she must have completed successfully the final year clinical trainings, the research paper must be accepted and should obtain recommendation of the department.

5.7.2.5.2 Examination:

The examination consists of written in the form of MCQ and essay, viva and practical clinical examination. A Candidate must score 70% and above in the aggregate results to qualify for award of the specialty certification. The grading shall be:

Written:	20%
Clinical:	20%
OSCE	20%
Viva:	10%
Past cumulative results	30%

Past cumulative is the aggregate of the following examination results and the periodic progressive assessments:

1. Year I exam --------20%
2. Progressive assessment---------------------10%

5.7.2.5.3 Examining Board:

External examiner chairs the examination board. At the end of the examination the board meets and decides on the final results of the examinations. The

external examiner has the final say. The results will be forwarded to MFAC for final approval.

5.7.2.5.4 resit Examination

Candidates who fail in the Final examination may appear for reseat examination as per the recommendation of the Examination Board.

5.7.2.3 Research

Research is one important component of the Department's instructional strategy. The research exercise is included in the curriculum with the intention to enable the trainee to undertake operational and clinical researches to investigate Emergency Medical problems and find out solutions. Faculties coach trainees in research activities. Each trainee will have at least one advisor. In addition trainees receive formal training on project development, appraisal and data management. This training will be conducted in collaboration with the department of public health.

The trainees are expected to produce at least one acceptable research paper to be eligible to sit for the final qualifying examination. The research proposals will be reviewed and amended before being carried out. Upon completion of the research the trainees are expected to defend their paper in presence of all faculties, trainees and other invited guests. After the necessary corrections are made finally the external examiner reviews the research products.

5.8 Resource Profile

5.8.1 Infrastructure

The department of EMS is located in Tikur Abessa Referral Teaching Hospital. The department will have lecture room for where the different teaching sessions are being conducted and a small library.

The department will utilizes three hospitals for teaching post and undergraduate students; namely Tikur Anbessa (where the department is based),St. Paul's and Zewditu Memorial hospitals. The hospitalswill be equipped to run postgraduate programs. Each hospital has operation rooms, outpatient and inpatients services, laboratory and other diagnostic facilities

2. In addition to existing staff the department has collaboration with the

following universities and their staff will involve in training by visits or video Conference.

1. Wisconsin university in Madison USA
2. Toronto university in Canada
3. George Washington University in Washington DC, USA
4. Witswatersr and university In Johannesburg, South Africa

5.8.2 Training Facilities

These are facilities available in the department and the medical school:

1.The department has emergency room which being equipped to acceptable level for the training and also provision of better patient care.

2.There is state of the art emergency medicine training center with all available equipments in the skills laboratory.

3.There are number of video conferencing facilities and teleconferencing unit in the medical school.

4.There is development process of the departments Library in addition to the main libraries of the medical school.

5.class rooms and other facilities in the medical school can be shared

Text books for reading

Tintinalli JE. Emergency Medicine: A Comprehensive Study Guide, 6th ed., New York, NY: McGraw Hill, 2007.

2. Harwood-Nuss AL. The Clinical Practice of Emergency Medicine, 3rd ed., Philadelphia, PA: Lippincott Williams and Wilkins, 2001.

3. Marx JA. *Rosen's Emergency Medicine: Concepts and Clinical Practice*, 5th ed., St. Louis, MO: Mosby, Inc., © 2007

Procedures Observed Done

1 Naso and Oro Tracheal ntubation

2 Cricothyrotomy

3 Cardioversion/Defibrilation

4 Tube Thoracostomy

5 Pericardiocentesis

6 Lumbar Puncture

7 Laceration Repair

8 Vaginal Delivery

9 Closed fracture splintting with or

i. without reduction

10 Dislocation reduction

11 Central vein access

12 Emergency bedside Ultrasound

14 Conscious sedation

13 Pre-Hospital Accompanying

15. Major Resuscitation

16. Intubation
