# Supplementary material for: Design and Implementation of a postgraduate curriculum to support Ethiopia's first emergency medicine residency training program: the Toronto Addis Ababa Academic Collaboration in Emergency Medicine (TAAAC-EM)
Source: BMC Med Educ. 2018 Apr 6;18:71. doi: 10.1186/s12909-018-1140-3 (PMC5889606; doi:10.1186/s12909-018-1140-3)
Supplement: Supplementary file 2 — Teaching Trip 1–12 Templates at a glance. Sample Detailed Teaching Template – Trip 3. (PDF 166 kb) [file 12909_2018_1140_MOESM2_ESM.pdf]

Appendix 2A: Teaching Templates 1-12 at a glance
Appendix 2B: Sample Detailed Teaching Template – Trip 3

APPENDIX 2A: Teaching Trip 1-12 Templates at a glance

TAAAC(EM) Teaching Trip #1: November 20____

	**Week 1**	**Week 2**	**Week 3**	**Week 4**
**Tues (Jr)**	Shock	Airway Management	Triage Principles (Admin 1)	Intro to Trauma Principles
**Wed (Jr)**	Intro to Clin Epi (Clin Epi 1)	Bias and Chance (Clin Epi 2)	Causation and Association (Clin Epi 3)	Airway Practical Workshop
**Thurs (Sr)**	ACS/Ischemia	CHF/Cardiogenic Shock	CRVHD/IE	Intro to Quality (Admin 2)
**Thurs Resident Seminar (All)**	Seizures	Upper Airway Emergencies	Tetanus/Botulism/MG	DVT/PE
**Thurs Practical Session (All residents except US session divided Jr/Sr)**	ECG Intro	Procedural Sedation (Adults)	Sim Lab	Ultrasound: FAST and IVC scanning

TAAAC(EM) Teaching Trip #2 : February 20____

	**Week 1**	**Week 2**	**Week 3**	**Week 4**
**Tues (Jr)**	Head Trauma	Spine Trauma	Chest Trauma	Cardiac Dysrhythmias
**Wed (Jr)**	Measures of Disease Frequency (Clin Epi 4)	Diagnostic Tests (Clin Epi 5)	Study Design: Overview and Experimental Designs (Clin Epi 6)	CT head interpretation
**Thurs (Sr)**	Intro to Toxicology	The Acute Abdomen	Upper GI Emergencies	ED Flow (Admin 3)
**Thurs Resident Seminar (All)**	Alcohol Emergencies	Cardiac Drugs Toxicology	ASA/Acetaminophen Overdose	Organophosphate and Rat Poison Overdose/Ingestion
**Thurs Practical Session (All residents except US session divided Jr/Sr)**	LP in Adults/Peds and Chest Tubes	Parascentesis, Thorascentesis, Pericardioscentesis	Sim Lab Cases	Ultrasound: 1st Trimester (IUP)

Appendix 2A: Teaching Templates 1-12 at a glance
Appendix 2B: Sample Detailed Teaching Template – Trip 3

TAAAC(EM) Teaching Trip #3: May 20____

	**Week 1**	**Week 2**	**Week 3**	**Week 4**
**Tues (Jr)**	Approach to Adult with Fever	Respiratory Infections	TB Emergencies	CNS Infections
**Wed (Jr)**	Non-Experimental Study Design (Clin Epi 7)	Systematic Reviews, Meta-analysis, & Guidelines (Clin Epi 8)	Asking a Good Research Question (Clin Epi 9)	CXR/AXR Interpretation
**Thurs (Sr)**	Anemia and Transfusions	Diarrhea and Lower GI Emergencies	Stroke	Patient Safety (Admin 4)
**Thurs Resident Seminar (All)**	Lightening and Electrical Injuries	Burns and Smoke Inhalation	Facial Trauma	Pelvic and Hip Fractures
**Thurs Practical Session (All residents except US session divided Jr/Sr)**	Upper Extremity Xrays	Upper Extremity Casting and Reductions	Sim Lab	Ultrasound: PTX/effusions and DVT

TAAAC(EM) Teaching Trip # 4: November 20____

	**Week 1**	**Week 2**	**Week 3**	**Week 4**
**Tues (Jr)**	Shock	Airway Management	Triage Principles	Intro to Trauma
**Wed (Jr)**	Clin Epi 1	Clin Epi 2	Clin Epi 3	Airway Workshop
**Thurs (Sr)**	HIV Emergencies	Approach to Psychiatric Patients in the ED	Endocrine Emergencies (excluding DM)	Pre-hospital Care (Admin 5)
**Thurs Resident Seminar (All)**	Renal Failure and Hyperkalemia	Electrolyte abnormalities (excluding hyperkalemia)	Diabetic Emergencies (incl. hypoglycaemia)	Coma/Delirium
**Thurs Practical (All residents except US session divided Jr/Sr)**	ECG Interpretation	Suturing and Tendon Repair	Sim Lab	Ultrasound: FAST and IVC scanning

Appendix 2A: Teaching Templates 1-12 at a glance
Appendix 2B: Sample Detailed Teaching Template – Trip 3

TAAAC(EM) Teaching Trip #5: February 20____

	**Week 1**	**Week 2**	**Week 3**	**Week 4**
**Tues (J)r**	Head Trauma	Spine Trauma	Chest Trauma	Cardiac Dysrhythmias
**Wed (Jr)**	Clin Epi 4	Clin Epi 5	Clin Epi 6	CT head interpretation
**Tues (Sr)**	OB Trauma	OB Emergencies	Approach to Gyne Emergencies (incl. PEP/GBV)	GI/GU trauma
**Thurs Resident Seminar (All)**	PIH, Pre-eclampsia, Eclampsia	1st Trimester Emergencies (incl. ectopics)	Septic Arthritis, Osteomyelitis, Arthritis	Approach to Headache
**Thurs Practical (All residents except US session divided Jr/Sr)**	Visual Stim Session: Optho	Visual Stim Session: ENT	Sim Lab	Ultrasound: 1st Trimester (IUP)

TAAAC(EM) Teaching Trip #6: May 20____

	**Week 1**	**Week 2**	**Week 3**	**Week 4**
**Tues (Jr)**	Adult with Fever	Respiratory Infections	TB Emergencies	CNS Infections
**Wed (Jr)**	Clin Epi 7	Clin Epi 8	Clin Epi 9	CXR/AXR interpretation
**Tues (Sr)**	Advanced Dysrhythmias	Wounds/Rabies/ Bites	Urological Emergencies	Altered Mental Status and Delirium
**Thurs Resident Seminar (All)**	Upper Extremity Fractures and Dislocations	Lower Extremity Fractures and Dislocations	Cranial Nerve Abnormalities	Approach to Neuropathies
**Thurs Practical (All residents except US session divided Jr/Sr)**	Peripheral Nerve Blocks UE and facial	Peripheral Nerve Blocks LE	Sim Lab	Ultrasound: PTX/effusion/DVT

Appendix 2A: Teaching Templates 1-12 at a glance
Appendix 2B: Sample Detailed Teaching Template – Trip 3

TAAAC(EM) Teaching Trip #7: November 20____

	**Week 1**	**Week 2**	**Week 3**	**Week 4**
**Tues (Jr)**	Shock	Airway Management	Triage Principles	Intro to Trauma
**Wed (Jr)**	Clin Epi 1	Clin Epi 2	Clin Epi 3	Airway Workshop
**Thurs (Sr)**	ACS/Ischemia	CHF/Cardiogenic Shock	CRVHD/IE/valvular heart disease	Intro to Quality (Admin 2)
**Thurs Resident Seminar (All)**	Asthma/COPD	Intestinal obstruction, Ileus & Constipation	Approach to Jaundice	STIs and Vulvovaginitis
**Thurs Practical (All residents except US session divided Jr/Sr)**	Visual Stim Session: Dermatology	ECG Interpretation	Sim Lab	Ultrasound: FAST/IVC

TAAAC(EM) Teaching Trip #8: Feb 20____

	**Week 1**	**Week 2**	**Week 3**	**Week 4**
**Tues (Jr)**	Head Trauma	Spine Trauma	Chest Trauma	Cardiac Dysrhythmias
**Wed (Jr)**	Clin Epi 4	Clin Epi 5	Clin Epi 6	CT head interpretation
**Thurs (Sr)**	Toxicology Cases	The Acute Abdomen	Upper GI Emergencies	ED Flow (Admin 3)
**Thurs Resident Seminar (All)**	Malaria	Hypertensive Emergencies	Typhoid, Dengue and Relapsing Fever	Soft Tissue Infections (Abscesses, tenosynovitis, ulcers)
**Thurs Practical (All residents except US session divided Jr/Sr)**	Soft tissue neck and facial xrays	Cervical, Thoracic and Lumbar Xray interpretation	Sim Lab	Ultrasound: 1st Trimester (IUP)

Appendix 2A: Teaching Templates 1-12 at a glance
Appendix 2B: Sample Detailed Teaching Template – Trip 3

TAAAC(EM) Teaching Trip #9: May 20____

	**Week 1**	**Week 2**	**Week 3**	**Week 4**
**Tues (Jr)**	Adult with Fever	Respiratory Infections	TB Emergencies	CNS Infections
**Wed (Jr)**	Clin Epi 7	Clin Epi 8	Clin Epi 9	CXR/AXR interpretation
**Thurs (Sr)**	Anemia & Transfusions	Diarrhea and Lower GI Emergencies	Stroke	Patient Safety (Admin 4)
**Thurs Resident Seminar (All)**	Thrombocytopenia and splenomegaly	Hand Injuries	Approach to Back Pain	Cholecystitis/Biliary Colic
**Thurs Practical (All residents except US session divided Jr/Sr)**	LE extremity xrays	LE casting and reductions	Sim Lab	Ultrasound: PTX/effusion/DVT

TAAAC(EM) Teaching Trip # 10: November 20____

	**Week 1**	**Week 2**	**Week 3**	**Week 4**
**Tues (Jr)**	Shock	Airway Management	Triage Principles	Intro to Trauma
**Wed (Jr)**	Clin Epi 1	Clin Epi 2	Clin Epi 3	Airway Workshop
**Thurs (Sr)**	HIV Emergencies	Approach to Psychiatric Patients in the ED	Endocrine Emergencies (excluding DM)	Pre-hospital Care (Admin 5)
**Thurs Resident Seminar (All)**	Renal Failure and Hyperkalemia	Electrolyte abnormalities (excluding hyperkalemia)	Diabetic Emergencies (incl. hypoglycaemia)	Coma/Delirium
**Thurs Practical (All residents except US session divided Jr/Sr)**	Introduction to ECG	Suturing and Tendon Repair	Sim Lab	Ultrasound: FAST and IVC scanning

Appendix 2A: Teaching Templates 1-12 at a glance
Appendix 2B: Sample Detailed Teaching Template – Trip 3

TAAAC(EM) Teaching Trip #11: February 20____

	Week 1	Week 2	Week 3	Week 4
**Tues (J)r**	Head Trauma	Spine Trauma	Chest Trauma	Cardiac Dysrhythmias
**Wed (Jr)**	Clin Epi 4	Clin Epi 5	Clin Epi 6	CT head interpretation
**Tues (Sr)**	OB Trauma	OB Emergencies	Approach to Gyne Emergencies (incl. PEP/GBV)	GI/GU trauma
**Thurs Resident Seminar (All)**	PIH, Pre-eclampsia, Eclampsia	1st Trimester Emergencies (incl. ectopics)	Septic Arthritis, Osteomyelitis, Arthritis	Approach to Headache
**Thurs Practical (All residents except US session divided Jr/Sr)**	Visual Stim Session: Optho	Visual Stim Session: ENT	Sim Lab	Ultrasound: 1st Trimester (IUP)

TAAAC(EM) Teaching Trip #12: May 20____

TRIP 6: MAY 20_______

	Week 1	Week 2	Week 3	Week 4
**Tues (Jr)**	Adult with Fever	Respiratory Infections	TB Emergencies	CNS Infections
**Wed (Jr)**	Clin Epi 7	Clin Epi 8	Clin Epi 9	CXR/AXR interpretation
**Tues (Sr)**	Advanced Dysrhythmias	Wounds/Rabies/ Bites	Urological Emergencies	Altered Mental Status and Delirium
**Thurs Resident Seminar (All)**	Upper Extremity Fractures and Dislocations	Lower Extremity Fractures and Dislocations	Cranial Nerve Abnormalities	Approach to Neuropathies
**Thurs Practical (All residents except US session divided Jr/Sr)**	Peripheral Nerve Blocks UE and facial	Peripheral Nerve Blocks LE	Sim Lab	Ultrasound: PTX/effusion/DVT

APPENDIX 2B: Sample Detailed Teaching Template

TAAAC-EM Teaching Trip #3: May 2015

The **junior AAU residents** (i.e. first year residents) will be given protected teaching on Tuesday, Wednesday and Thursday afternoons.

The **senior AAU residents** (second and third year residents) have protected teaching on Thursday afternoons. However, if their clinical duties allow, they are welcome to attend the Tues and Wed afternoon sessions as well.

Journal Club will be held on the last Friday of the month.

Note:
Mondays, Fridays, and morning half-days on Tuesday, Wednesday and Thursday will be dedicated to clinical bedside teaching in the ED for those rotating in the ED.

Most of your required readings are from Tintinalli 7th edition. For supplementary readings please refer to your handout. The handout will be given to you at the beginning of week one.

TEMPLATE AT A GLANCE:

	**Week 1**	**Week 2**	**Week 3**	**Week 4**
**Tues (Jr)**<br>**TBD; AAU staff**	Approach to Adult with Fever	Respiratory Infections	TB Emergencies	CNS Infections
**Wed (Jr)**	CXR/AXR Interpretation	Non-Experimental Study Design<br>(Clin Epi 7)	Systematic Reviews, Meta-analysis, & Guidelines<br>(Clin Epi 8)	Asking a Good Research Question<br>(Clin Epi 9)
**Thurs (Sr)**	Anemia and Transfusions	Stroke	Diarrhea and Lower GI Emergencies	Patient Safety<br>(Admin 4)
**Thurs Practical Session**<br>**(All residents except US session divided Jr/Sr)**	Upper Extremity Xrays	Upper Extremity Casting and Reductions	Sim Lab	Thorascentesis/<br>Parascentesis/<br>Pericardioscentesis

Appendix 2A: Teaching Templates 1-12 at a glance
Appendix 2B: Sample Detailed Teaching Template – Trip 3

DETAILED TEMPLATE WEEK 1-4:

**WEEK 1**	**TUESDAY (Juniors 1:30-3:00pm)**	**WEDNESDAY (Juniors 1:30-3:00pm)**	**THURSDAY (Seniors 12:30-1:30pm)**	**THURSDAY PRACTICAL SESSION (ALL 2:30-4:30pm)**
	**Topic**: Adult with fever<br><br>**Readings**:<br>Tintinalli 7th edition chapter 156 (p 1082-1088)<br><br>**Objectives**:<br>1. To generate differential diagnosis of the adult with fever<br>2. To discuss work-up and testing	**Topic**: Chest xray and abdominal xray interpretation<br><br>**Readings**: None<br><br>**Objectives**:<br>1. Approach to Chest Xray<br>2. Approach to Abdominal Xray	**Topic:** Anemia and transfusions<br><br>**Readings:**<br>**One of:**<br>Rosen's Chapter 7<br>**OR**<br>Chapter 223 In Tintinalli<br><br>**Objectives:**<br>1. To review updated thresholds for treatment of anemia in the Emergency department.<br>2. To review modalities and complications of current transfusion practice in Ethiopia.	**Topic:** Upper extremity xrays<br><br>**Readings:** Suggested for this session, but important for the casting session in week 2. Tintinalli's Emergency Medicine 7th ed. Chapters 264 (Initial Evaluation and Management) sections on upper extremity injury. If time permits read all of Chapters 265, 266, 267 with a focus on fracture management<br><br>**Objectives:**<br>1.Develop an approach to the interpretation of upper extremity x-rays<br>2.Understand pediatric differences<br>3.Interpret abnormal xrays

Appendix 2A: Teaching Templates 1-12 at a glance
Appendix 2B: Sample Detailed Teaching Template – Trip 3

**WEEK 2**	**TUESDAY (Juniors 1:30-3:00pm)**	**WEDNESDAY (Juniors 1:30-3:00pm)**	**THURSDAY (Seniors 12:30-1:30pm)**	**THURSDAY PRACTICAL SESSION (ALL 2:30-4:30pm)**
	**Topic**: Respiratory infections<br><br>**Readings**:<br>Tintinalli's Emergency Medicine, 7th Ed. Chapters 57 (CHF), 65 (Respiratory Distress), 68 (CAP/Aspiration, 69 (Empyema/Lung Abscess), 70 (TB), 71 (Pneumothorax), 72 (Asthma), 73 (COPD)<br><br>**Objectives**:<br>1.Focus on causes of dyspnea in the ED<br>2.Review of infectious causes of dyspnea<br>3.Review of non-infectious causes of dyspnea<br>4.Approach to other common respiratory presentations in the ER	**Topic**: Clinical epidemiology 7: Non-experimental study design<br><br>**Readings**:<br>Fletcher p. 81-84, 91-100, 116-118, 194-195, p 64 and 70<br><br>**Objectives**:<br>1. Understand the strengths and limitations of basic non-experimental designs	**Topic:** Stroke<br><br>**Readings:**<br>Tintinalli's Emergency Medicine Manual, 7th Edition, Chapter 141<br><br>**Objectives:**<br>1. Define ischemic and haemorrhagic stroke<br>2. Identify risk factors contributing to stroke<br>3. Understand physical examination and CT scan findings in stroke<br>4. Discuss urgent management of stroke<br>5. Outline outpatient management after stroke	**Topic:** Upper extremity casting and reductions<br><br>**Readings**:<br>Tintinalli's Emergency Medicine 7th ed. Chapters 264 (Initial Evaluation and Management) sections on upper extremity injury. If time permits read all of Chapters 265, 266, 267 with a focus on fracture management<br><br>**Objectives**:<br>1. Perform focused assessments of fractures and associated neurovascular injuries<br>2. Practice reduction techniques<br>3. Practice common splinting techniques<br>4. Consolidate radiography interpretation skills

Appendix 2A: Teaching Templates 1-12 at a glance
Appendix 2B: Sample Detailed Teaching Template – Trip 3

**WEEK 3**	**TUESDAY (Juniors 1:30-3:00pm)**	**WEDNESDAY (Juniors 1:30-3:00pm)**	**THURSDAY (Seniors 12:30-1:30pm)**	**THURSDAY PRACTICAL SESSION (ALL 2:30-4:30pm)**
	**Topic:** TB emergencies<br><br>**Readings:**<br>Tintinalli 7th edition Chapter 70 p494-500<br><br>**Objectives**:<br>1. Short review of PTB and EPTB in Ethiopia (clinical presentation, diagnosis and treatment)<br>2. Acute presentations and management of PTB and EPTB in the ED<br>3. Treatment complications of TB<br>4. Treatment complications of TB/HIV patients	**Topic**: Clinical Epidemiology 8: Special designs, systematic reviews, meta-analysis and guidelines<br><br>**Readings**:<br>Fletcher p 205-218, p2-3, p 161<br><br>**Suggested Reading:**<br>JAMA 273(16) p 1292-1295 and 273(20)p1610-1613<br><br>**Objectives**:<br>1.To understand the key elements of systematic reviews and the potential uses for systematic reviews<br>2.To introduce health services and qualitative research	**Topic**: Diarrhea and Lower GI emergencies<br><br>**Readings**:<br>Tintinalli 7th edition Chapters 74, 76, 77, 79, 84, 85, 86, 87, 88 in Tintinalli<br><br>**Objectives**:<br>1.Identify the general causes of abdominal pain in the ED<br>2.Review causes of lower GI bleeding, and develop treatment and management strategies<br>3.Review IBD, and have an approach to emergency tx and management<br>4.Identify and tx surgical emergencies such as bowel obstructions, volvulus, mesenteric ischemia, appendicitis, bowel perforations etc<br>5.Develop an approach to the diagnosis and management of diarrhea in the ED	**Topic:** Simulation Cases

Appendix 2A: Teaching Templates 1-12 at a glance
Appendix 2B: Sample Detailed Teaching Template – Trip 3

**WEEK 4**	**TUESDAY (Juniors 1:30-3:00pm)**	**WEDNESDAY (Juniors 1:30-3:00pm)**	**THURSDAY (Seniors 12:30-1:30pm)**	**THURSDAY PRACTICAL SESSION (ALL 2:30-4:30pm)**
	**Topic**: CNS Infections<br><br>**Readings**:<br>Tintinalli 7th edition Chapter 168<br><br>**Objectives**:<br>1.Review etiology, pathophysiology, treatment and ED management of bacterial and viral meningitis and encephalitis<br>2.To determine causes, pathophysiology, ED dx and tx of brain abscesses	**Topic**: Clinical Epidemiology 9: Asking a good research question<br><br>**Readings**:<br>Fletcher p2-3, p 161<br><br>**Objectives**:<br>1.To introduce quantitative decision making, health services research and qualitative research:	**Topic:** Admin 4: Patient Safety<br><br>**Readings:**<br>Beyond the organizational accident, Quality and Safety in HealthCare, 2004;13 Supplement 2, ii28-33<br><br>**Objectives:**<br>1. To understand what "unsafe act" and error is<br>2. To understand why errors occur<br>3. To gain an understanding of human factors engineering and systems in patient safety<br>4. To understand how teamwork and communication play a role in patient safety	**Topic**: Paracentesis, Thoracentesis and Pericardiocentesis<br><br>**Readings:** Tintinalli 7th Edition pg. 603-604, pg. 472-473, Chapter 37<br><br>**Objectives:**<br>1. To understand the indications and contraindications for performing paracentesis, thoracentesis and pericardiocentesis procedures<br>2. Understand the relevant anatomy and procedural steps for performing paracentesis, thoracentesis and pericardiocentesis<br>3. Feel comfortable interpreting paracentesis, thoracentesis and pericardiocentesis results where applicable<br>Understand the role of bedside US in performing paracentesis, thoracentesis and pericardiocentesis safely
