# Supplementary material for: Design and Implementation of a postgraduate curriculum to support Ethiopia's first emergency medicine residency training program: the Toronto Addis Ababa Academic Collaboration in Emergency Medicine (TAAAC-EM)
Source: BMC Med Educ. 2018 Apr 6;18:71. doi: 10.1186/s12909-018-1140-3 (PMC5889606; doi:10.1186/s12909-018-1140-3)
Supplement: Supplementary file 3 — Brief Explanation of CanMEDS/EthioMEDS roles. (PDF 84 kb) [file 12909_2018_1140_MOESM3_ESM.pdf]

Appendix 3: Brief Explanation of CanMEDS/EthioMEDS roles

EthioMEDS are derived from CanMEDS roles.
In 1996, the Royal College of Physicians and Surgeons of Canada (RCPSC) Council adopted a new framework of core competencies for all specialists, called the "CanMEDS Roles" a derivative from "Canadian Medical Education Directives for Specialists". This framework of core competencies, expected and required for residents in every residency training program in Canada, includes the Roles of **Medical Expert (the central role), Communicator, Collaborator, Manager, Health Care Advocate, Scholar and Professional.** This now forms the basis of the standards of the educational mission of the Royal College. It has been incorporated into accreditation, evaluation and examinations, as well as objectives of training and standards, for continuing professional development. Furthermore, the CanMEDS framework of competencies that began as an initiative of forward-thinking Fellows of the RCPSC and family physician contributors has now become a popular standard for medical education in Canada and around the world. http://rcpsc.medical.org/canmeds/index.php

Brief explanation of EthioMEDS roles:
Like Canada, Ethiopia has its own challenges to meet if it is to provide health care to its population. As a low income country Ethiopia has identified a need to expand and reform its health services. At present there is a significant gap between resources and the need for medical/surgical care. In order to address this treatment gap it is necessary that Ethiopian postgraduate medical/surgical residents are trained and acquire expertise in the multiple roles required for the task. With this in mind, the Ethiopian residency training programs in the Faculty of Medicine, Addis Ababa University have begun to develop 5 mandatory roles for resident acquisition during training. These are modified from the CanMEDS roles to **Clinical Expert, Leader, Educator, Scholar and Advocate**. These have now become incorporated into the objectives of training and into the routine residency evaluations and examinations, and they are now directing the standards for continuing professional development.

Description of each EthioMEDS role

Clinical Expert
- demonstrate diagnostic and therapeutic skills for ethical and effective patient care
- access and apply relevant information to clinical practice
- demonstrate effective consultation services with respect to patient care, education and legal opinions

Leader
- demonstrates skills as a *collaborator*, contributing effectively with other health care professionals
- allocates and utilizes and *manages* finite resources effectively and wisely
- deliver highest quality care with integrity, honesty and compassion
- exhibit appropriate personal and interpersonal *professional* behaviours
- practise medicine ethically consistent with obligations of a physician

Educator
- demonstrates effective skills as a *communicator* with patients and their families
- teaches and imparts knowledge and skills to patients, their families, staff, agencies and gov'nt as required.
- facilitates the education of patients, housestaff/students and other health professionals

Scholar
- develops, implements and monitors a personal continuing education strategy
- critically appraise sources of medical information and contributes to the development of new knowledge

Advocate
- identify the important determinants of health affecting patients
- contributes effectively to identify the important determinants of health affecting patients in order to improve the health of patients and communities
- recognize and respond to those issues where advocacy is appropriate
